# The Application of Intermittent Filtration to Domestic Filters1Read at the annual meeting of the Buffalo Academy of Medicine, June 23, 1894.

**Published:** 1895-03

**Authors:** George W. Rafter

**Affiliations:** Rochester, N. Y.


Buffalo Medical and Surgical Journal

Vol. XXXIV. MARCH, 1895. No. 8.

### Original Communications.

### THE APPLICATION OF INTERMITTENT FILTRATION TO DOMESTIC FILTERS.[1]

By GEORGE W. RAFTER, Rochester, N. Y.

If one were to propose to an association of practical water works managers as a question for discussion, which is more important—whether to secure for large cities an abundant supply of water of medium quality, that is, a supply which may at times be bad, or a restricted supply of absolutely unexceptionable quality, that is, a supply which will always be good—we would find, I think, the consensus of opinion decidedly in favor of the abundant supply of medium quality.

If, however, we propose the same question to an association of hygienists, we will probably be told that as regards standards of purity of public water supplies nothing is admissible which can by any possibility contain the germs of infectious disease ; and while we may tacitly accept this latter as the final conclusion of recent scientific endeavor, we, nevertheless, find ourselves, when we attempt to obtain a public supply for a large city which answers to this requirement, confronted with the stern fact that, aside from small quantities of sparingly distributed spring water, practically no such supplies exist.

We learn, then, at the very outset, that in the selection of the great majority of public water supplies there must be a compromise as regards quality, and it is largely due, without doubt, to this nearly universal rule the idea has become so generally prevalent that the first consideration should be abundance, quality only entering into the account when the equation of quantity is fully satisfied. Further, if we examine the matter in detail we shall

1. Read at the annual meeting of the Buffalo Academy of Medicine, June 23, 1894.

find that in selecting the water supplies of most of our American cities and towns, quantity has thus far been the sole consideration governing; and that, other than remotely, quality has not been considered at all.

Moreover, even when we threw quality largely out of the account, the choice is not always easy because of great expense, and when we assume, as the teachings of hygiene show that we should, certainly medium quality, the choice even then frequently becomes a matter of extreme difficulty.

A number of years ago the present speaker had occasion as a part of his ordinary duties to examine somewhat in detail every possible source from which either a temporary or permanent supply of potable water could be drawn for the city of Rochester. In the course of the study something like eighteen distinct sources were examined, with the result of showing that taking into account everything, the choice was really narrowed to Hemlock lake, the source formerly selected, which, while admittedly a source of inexceptionable quality, was still, in the opinion of many citizens of Rochester, hardly available as an additional supply by reason of the great distance (thirty miles) which the water must be transported.

The result of a fairly exhaustive examination was to show, however, that taking into account quality as well as cost of obtaining a given quantity, it followed that Hemlock lake, even though thirty miles distant, was by far the preferable source of supply for the city of Rochester.

Western New York, looked at casually, would be considered a well-watered region, and since making the examination in question it has always seemed to me an exceedingly interesting fact that the repeated selection of Hemlock lake as the natural source of the potable water supply of the city of Rochester, by all the engineers who have examined the matter in detail since about 1860, when it was first proposed, down to the present, should show clearly that potable water of high quality and in large quantity is in reality rather a scarce commodity in Western New York. At any rate, we may draw the conclusion that the theoretical hygienic standard is in many localities practically impossible of attainment.

The foregoing may be taken, then, as indicating—albeit rather rapidly—that there are as yet but few public supplies, the water of which can be safely used for drinking without preliminary purification of some sort.

The art of filtration has now so far developed that the indispensable preliminary purification can be certainly obtained in every case where municipalities are willing to meet the additional expense, but it must be understood that thus far only a few American towns have shown themselves willing to provide this remedy for the entire supply. The reasons for this apathy may be found mostly in (1) a lack of appreciation of the importance of furnishing drinking water of the hygienist's standard; and (2) the considerable expense of filtering the whole supply for the purpose of furnishing drinking water, which naturally is only a very small proportion of the whole. Hence it results that practically the only way safe drinking water can be obtained from the average American public supply is by the use of domestic filters, wherein the drinking water of each family may be purified as used from day to day.

That there is a real demand for an efficient domestic filter, one that can be relied upon to furnish absolutely pure water at all times, to sterilize it in short, may be sufficiently illustrated by considering that since 1880 about 1,000 patents have been issued from the United States Patent Office for domestic filters alone. Nearly all of these are not filters in any proper sense of the word, but are merely strainers, and possess the defect of either clogging or else failing to purify at all.

In view of the demand for a good filter, the fact that only one or two of these have come into even moderate use is very satisfactory proof of lack of the essential qualities of a good filter in nearly the whole series.

Broadly, we may classify filters as either continuous or intermittent.

In continuous filters, water is passed through the filter in a continuous stream, the rate of flow depending, in many cases, upon the personal whim of the designer, and in others upon natural limitations imposed by the filtering material. With sand filters operated continuously, experience has indicated a certain rate beyond which it is impossible to operate and secure any special improvement of the effluent.

In intermittent filters the water is passed through the filtering material intermittently—that is, with intervals between each application of water. In such filters the material is preferably coarse, clean mortar sand, and the intervals of application are made long enough to insure the sinking of each dose of water below the surface of the sand before another application is made. In continuous filters, on the other hand, nearly every possible variety of material has been used, as, for instance, sand, gravel, charcoal, felt, porous stone, porous porcelain and many others; but at present sand, charcoal, porous porcelain and porous natural stone are the ones in more general use.

In order to understand the conditions essential for a perfect filter we need to consider briefly (1) the nature of the impurities to be got rid of by filtration; and (2) the rationale of the filtering operation itself.

Throwing out of account mineral impurities which are not, so far as effect on health is concerned, generally speaking, serious in any water likely to be selected as the source of a public supply, we may classify the ordinary impurities as: (1) Suspended impurities, which may be taken to include all miscellaneous particles of matter, either vegetable, animal or mineral, which have gained access to the water together with the minute forms of microscopical life, such as the algae, infusoria, rotifera, entomostraca, etc.; and (2) the dissolved impurities, including the nitrogenous products of decomposition; (3) disease germs—as for instance the germs of cholera, typhoid fever, and the like.

In a general way we may say that a water is satisfactory from the points of health, taste and comfort: (1) When it is free from suspended matter, both animal and vegetable; (2) when it is either colorless or nearly colorless; (3) when it contains enough dissolved oxygen to render it bright and refreshing; (4) when it contains not only no disease germs but also nothing which can by any possibility serve as a nourishment for such germs should any by chance gain access.

The perfect filter must be so arranged, then, as:

(1) To remove all suspended matter.

(2) To render a brown-colored water either colorless or nearly colorless.

(3) To furnish an effluent containing, at any rate, nearly the normal amount of dissolved oxygen.

(4) To furnish an effluent containing neither disease germs nor the nutrient food of such germs.

As a final condition of a perfect domestic filter, we may lay down the proposition that it must be so designed as to do its work efficiently for a long time without any attention. In the great majority of private houses there is no one sufficiently skilled in the theory and practice of filtration to give a filter such daily attention as most of those now in common use demand. The perfect filter must, therefore, be self-cleansing and automatic. Anything short of this is not in line with the best recent thought on domestic filtration.

We may now examine the theory of filtration a little in detail.

In the first place, it has been known for a long time that when impure water is poured upon porous ground at intervals, and in not too great quantities at any one point, it is not only quickly absorbed, but is found when flowing out, as from springs at lower levels, to be more or less completely freed from the organic impurities originally contained. This is Nature's process of purification, and in those localities where the proper materials exist naturally, as for instance in extensive sand areas, the water of the region, howsoever impure it may have been naturally, is rendered absolutely pure after filtering for a short distance through the ground. So marked is this action, that ordinary city sewage, when intermittently applied, may be so far purified by filtration through only five feet of clean sand, as to be indistinguishable by either chemical or bacteriological tests, from what are in most places considered to be unexceptionable sources of water supply. The present speaker has on several occasions drunk of the effluent from such sewage filters, so far as he knows, absolutely without ill effect.

The old idea of a filter was that of a strainer purely, but as the result of recent advances we now know that the purifying action of the soil in the case just considered is due to several distinct processes, as for instance:

(1) Simple filtration (*i. e.*, straining) or the separation and retention of the suspended matter.

(2) The precipitation and retention of the various organic substances in solution.

(3) The oxidation of the organic matter so arrested and retained, by the action of living organisms.

This latter step involves what is known as the process of nitrification, wherein two living organisms, which are specially designated as the nitrous and nitric organisms, absolutely resolve, when the proper conditions obtain, the objectionable organic matter of water into innocuous forms. Moreover, the nitrifying organisms are inimical to disease germs and other forms of bacterial life. It, therefore, follows that any water which has been thoroughly exposed to the nitrifying action is not only freed of bacteria—

sterilized—but has also been freed, by the same operation, of bacterial food. If, then, such water should, after filtering, become accidentally contaminated we may expect—what has indeed been found by experiment to be the case—that the new growth thus introduced will be relatively less luxurious than it would be in the effluent of any mere straining filter where dissolved organic matters are presumably mostly not removed.

As another attribute of the perfect filter we may say, then, that it must be so arranged as to permit the nitrifying organisms to do their work of reducing the organic matter of the treated water. By way of showing how this may be brought about, let us consider the present state of information in regard to nitrification.

As long ago as 1862, Pasteur, having in mind that fermentation was due to the action of living agents, advanced the idea that possibly the production of nitric acid in soils might also be due to a similar agency. Again, in 1873, A. Müller remarked that while the ammonia of sewage often changed rapidly into nitric acid, nevertheless solutions of ammonia and urea prepared in the laboratory remained unaltered. Müller suggested a ferment which was absent from the pure solutions prepared in the laboratory, but made no attempt to verify this by any experimental determination ; and it was not until 1877 that Schlössing and Müntz established the true nature of nitrification. In their paper published in that year they demonstrated by a series of brilliant experiments that nitrification must be the work of a living organism.

The next step was to discover and isolate the nitrifying organisms, and although Schlössing and Müntz fully recognized the importance of accomplishing this and materially advanced our knowledge of nitrification, still it is doubtful if they ever worked with really pure cultures. Certain difficulties of technique came in, which it is not necessary to refer to here, but which still delayed the final demonstration until 1890, in which year Dr. P. F. Frankland, in England, and Winnogradsky, in Switzerland, both announced the discovery of and described organisms capable of oxidizing ammonia to nitrates. At about the same time Warington, in England, and the biologists of the Massachusetts State Board of Health at Boston also succeeded in obtaining pure cultures of these organisms.

Without pursuing these interesting experimental researches at greater length, we may now state that the ascertained facts indicate that nitrification takes place in two stages, each characterized by a distinct organism. The office of one of these is to convert ammonia into nitrite; while the other converts nitrite into nitrate. Hence we have the nitrous and nitric organisms or ferments. Both are present in ordinary soils in enormous numbers; they are also present in natural waters and in sewage, where they quickly develop under certain conditions in quantity.

The conditions of success in nitrification, so far as ascertained, are:

(1) The presence of the natural food of the organisms.

(2) The presence of a sufficient supply of oxygen. The vast importance of this condition upon filtration will appear as we proceed.

(3) The presence of a salifiable base with which the nitric acid when formed may combine.

(4) A favorable temperature of the materials to be nitrified. Experiments show that nitrification is most active between the temperatures of 55° F. to 95°. Above 95° it diminishes rapidly until at about 130° F. it ceases entirely. Below 55° it diminishes, but does not entirely cease until about 32° is reached. As regards domestic filtration, the usual temperature of about 70° at which houses are kept may be considered, then, a very favorable one, as insuring the activity of the nitrifying organisms at all seasons of the year.

(5) Nitrification takes place best in the dark.

With the necessary conditions of nitrification before us we see at once why all sand filters worked continuously are of necessity foredoomed to failure. At the best such filters can only act as strainers with the result of either soon clogging up and entirely ceasing to work until cleaned, or else the inter-spaces become so filled with the organic matter strained out as to furnish breeding places for any bacteria present in the water, until, as has actually been found to happen, such filters often send out an effluent containing more bacteria than in the original water. The reason for this is that continuous filtration violates that fundamental principle of nitrification which affirms the presence of a considerable quantity of oxygen as an indispensable condition; hence, the working of any filter continuously is likely to lead, by an accumulation of organic matter in the interstices of the filter, to an aggravation of the evil which filtration is intended to correct.

It is, of course, true that all natural waters contain a certain quantity of dissolved oxygen, and to the extent of the influence of this there may be some slight nitrification in continuous filtration. Experience, however, has shown that the dissolved oxygen alone is insufficient to produce anything like an efficient resolution of the contained organic matter. And we may, therefore, take it as settled that intermittency of application is one of the indispensable conditions for complete nitrification.

The theoretically perfect filter will, therefore, be one embodying arrangements for supplying water in the proper quantity intermittently and at the same time insuring a sufficient supply of oxygen. The nitrifying temperature and absence of light are matters of detail easily attained and which need not be specially described here.

The filtering material will be clean, sharp sand resting upon gravel supported at the bottom of a filter barrel. The sand should be arranged in graduated layers progressively finer from above downwards. A layer of loam may be introduced a few inches from the top, although such a layer is not necessary for the success of such a filter. The chief office of the loam would be to introduce the nitrifying organisms, although now that we are able to cultivate them artificially they may be introduced at will to all parts of the filter and consequently the layer of loam becomes less necessary than before the methods of artificial propagation of the nitrifying organism were worked out.

Such a filter operated intermittently will perpetually resolve nitrogenized substances into:

(1) Free nitrogen gas, which passes into the common stock in the atmosphere; and

(2) By combination with oxygen to form nitric acid, which again enters into combination as rapidly as formed with mineral bases, such as lime, etc., which are always present in water, thereby producing harmless, soluble mineral nitrates, which are dissolved as fast as formed and pass off in the effluent.

The mineral nitrates, in any quantity, resulting from the nitrification of the organic impurities of potable waters are not objectionable to human beings, nor are they in any degree nutrient to bacterial life.

A filter of this character will also be fairly self-cleansing—that is, it will tend to keep itself perpetually clear of any accumulation of organic matter in the interstices of the filter; and, inasmuch as the property of self-cleansing is in reality a distinguishing feature of intermittent filters, we may properly, even at the risk of some little repetition, define at this point the conditions for self-cleansing, namely :

(1) A filter composed in its upper layers of coarse, porous filtering material, as for instance coarse mortar sand, with finer material below.

(2) Intermittency of application of water to such a filter.

(3) The presence in the voids of the filter of the nitrifying organisms in conjunction with a mineral base.

(4) For the most perfect results a temperature of about 70° F.

(5) The application of the water in such limited quantities as to furnish an opportunity for the nitrifying organisms to come in contact with every particle of water passing through the filter.

(6) In the case of water carrying silt in suspension, the provision of a silt-arrestor, through which the water passes before going upon the filter proper.

In regard to condition (3), it may be again stated that nitrifying organisms not only exist naturally in soils, but in the waters of all streams, ponds, lakes, as well. If we make an intermittent filter of clean, coarse sand, etc., as described, and apply any natural water, we find the nitrification nearly nothing in the beginning, but after a time gradually increasing, until finally a maximum rate of nitrification will be attained and, with uniform temperature, maintained indefinitely. The cultivation of the nitrifying organism in pure cultures is now a simple matter and such a filter may, therefore, be brought to a maximum state of efficiency (in less time than by the natural method) by the introduction of artificial cultures of the nitrifying organism, which becoming quickly established upon the surfaces of the sand grains, will produce complete nitrification, the same as ensues as the final result of the slower process of natural inoculation already described.

Such a filter is in the fullest sense of the word self-cleansing. A trial plant which has been used at Lawrence, Mass.,[1] continuously for five years filtering city sewage at the rate of about 2.3 gallons per square foot of area per day, still shows the sand clean below the first six inches ; it is the opinion of those who have watched the process from day to day that the renewal of the upper six inches on the filter in question will again make the filter practically as good as at the beginning. In filtering ordinary waters where the amount of organic matter to be resolved is far less than in city sewage, the daily quantity of water treated may be proportionately

1. At the Experiment Station of the Massachusetts State Board of Health.

greater, or as a general statement, subject to exception in some cases, we may say that ordinary potable waters may be filtered at the rate of about twenty-five gallons per square foot of filter area per day and yield a sterile effluent.

The following are a few results obtained at Lawrence, from tests in which different hardy varieties of bacteria have been added to water before filtering through an intermittent filter of the kind here described:

(1) *Bacillus Prodigious.*—A harmless but exceedingly hardy variety of bacteria was added in large quantity and the water filtered at the rate of forty-five gallons per square foot per day. The effluent came through absolutely sterile.

(2) *Bacillus Coli Communis.*—A common, hardy, intestinal form, universally present in domestic sewage, was applied in large quantities to a filter, filtering at the rate of forty gallons per square foot of area per twenty-four hours. Result: Absolutely sterile effluent.

(3) *Bacillus Typhosus.*—The bacillus of typhoid fever was added in large quantities and the water filtered at about a rate of fifty gallons per square foot per day, with a result, as in the previous case, of absolute removal.

As the result of a large amount of experimentation extending over a period of five years, it has been further fully determined that any given filter has a certain maximum rate of work beyond which it is impossible to force it, with a given water, and at the same time secure a complete removal of bacteria. Again, the slower the rate of filtration, the more certain we are to obtain absolute sterilization. In order, therefore, to keep within such bounds as will certainly insure perfect results always, we lay down the general rule that the rate of filtration should not exceed about twenty-five gallons per square foot of area per day. Very impure waters may be filtered up to or near to this limit and still yield a sterile effluent, while, on the other hand, fairly pure waters may, as indicated, be safely filtered at a considerably higher rate.

In order to illustrate the relation of rate of filtration to degree of purification attained, we will briefly consider another phase of the matter. With a filter five feet in depth and one square foot in area the total cubic contents is five cubic feet, equivalent to 37.5 United States gallons. The voids in the sand will equal about 40 per cent. of the whole volume, or about fifteen gallons. If filtering at the rate of fifteen gallons per day, the amount of water added in that time would just equal the volume of the voids. If filtering at the rate of forty-five gallons per day, the volume added would be three times that of the voids. The effect of increasing the rate upon the length of time the treated water would remain in contact with the minute nitrifying organisms distributed throughout the voids and clinging to the surfaces of the sand grains, is so obvious, therefore, as to require no further discussion.

We may also consider for a moment the real effect of the intermittency of application. In doing this, let us assume by way of illustration that water is applied to such a filter in half-gallon doses at the rate of one gallon per hour. We have then a depth of 0.8 of an inch of water thrown upon the surface of the filter every half hour. With coarse, upper sand this quantity will ordinarily disappear beneath the surface in a few minutes. As it gradually sinks into the sand it spreads itself in thin laminæ over the sand grains and at the same time the air is drawn into the space above while water is forced from the bottom. The process of gradual sinking into the inter-spaces of the filter and outflow of effluent from the bottom goes on until, at the end of a half hour, another dose of water is suddenly applied to the depth of 0.8 of an inch over the entire area of the filter. The air which has passed into the voids, following the downflow into the sand of the previous dose, is mostly retained therein and forced forward through the filter. We have then alternate layers of air and water passing down through the voids, thereby insuring not only thorough mechanical oxygenation of the water, but absolute contact of every particle with the nitrifying organisms in the voids. The movement of the water through the sand even with comparatively high rates of filtration is seen to be quite slow.

The mechanical arrangements for effecting the various operations here described are so clearly shown in the accompanying sketches as to render extended description unnecessary.

In conclusion it may be stated that there is no reason why, after the intermitting apparatus of one of these filters is properly adjusted, it may not go on furnishing sterile water without any especial attention for several years. Even at the end of that time the only attention required would probably be a mere renewal of the upper layer of sand; at any rate such results may be expected with all waters which are tolerably free of silt. With silt-bearing or muddy waters, the silt catcher would, of course, require occasional attention.

Another point which may be properly noticed is in relation to just the difference between intermittent filtration and continuous filtration through sand, viz.:

In continuous filtration, water is supplied to the upper surface of the filter in large quantities and flows continuously into the voids, completely filling them. Air is, therefore, excluded and the process becomes mostly one of mere straining. The organic matter accumulates in the voids of the filter and in a short time seriously limits its action, precisely as occurs in the Pasteur filter and in all other filters of the straining class. In an intermittent filter, the nitrifying organisms are aërobian forms; that is, they require the presence of oxygen as a necessary condition for their growth. The exclusion of atmospheric air in the continuous process soon results, then, in cessation of active nitrification, although the process may go on for some time because of the presence of a small amount of dissolved oxygen in all natural waters. The relations of continuous filtration to intermittent and the consequent variations in the degree of nitrification attained in the same filter, have been observed in the series of experiments at Lawrence, already referred to, and the conclusion reached that with sand filters used continuously, the inert organic matter and living organisms both tend to permanently increase, until finally the inter-spaces become entirely filled. If any disease germs are present in the treated water, the filter may become the source of the greatest danger. Indeed, cases have actually occurred in which the bacilli of typhoid fever have so far increased in the inter-spaces of a continuous filter as to furnish many thousands in every glassful. As emphasizing the difference between intermittent and cantinuous sand filters, we may say, in a few words, that intermittent filters destroy disease germs when present in the treated water, while continuous filters, by furnishing a nidus, may lead to their extensive multiplication.

Reference has been made in the foregoing to the effect of filtration on reduction of color in brown-colored waters. On this point it may be said that the Lawrence experiments indicate, with filters five feet in depth, a reduction of color of about 50 per cent. by intermittent filtration. This would so far improve ordinary waters as to render them unobjectionable. The experiments indicate, however, more complete removal of color with deep filters than with shallow, and where very brown-colored, peaty waters are encountered, a greater depth of filter than five feet can possibly be used with good effect.

As to the amount of dissolved oxygen in the effluents from intermittent filters, the experiments show from 90 to 100 per cent. of that necessary for complete saturation at the actual temperature.

Reference may be made to the Pasteur-Chamberland filter, invented by Dr. Chamberland in the laboratory of the great French chemist, Louis Pasteur. This filter is a strainer purely, but when skilfully handled it may be made to yield a sterile effluent. The filtering material is a thin tube of porous porcelain, through which water is forced by pressure. With water containing any amount of impurity the outside of the tube soon becomes covered with a film, composed of the organic or mineral impurities which are naturally present in the water and which, if not frequently removed, soon clog the filter, entirely preventing further action until the tube is cleaned. Again, after the tube has been several times fouled, any bacteria present in the treated water penetrate the pores of the filter tube itself and finally pass through, appearing in the effluent. When this occurs, the tube must be purified by heat before it is again capable of yielding sterile water. In order to understand how bacteria may finally pass through the Pasteur tube we have only to consider that the porosity of the tube is perhaps equivalent to one micron or say the one twenty-five thousandth part of an inch. Many bacteria, as for instance the typhoid bacillus, are not more than one-fifth of a micron or one one-hundred thousandth of an inch in diameter, while typhoid spores (if they exist) are even much less than this. As a mere matter of mechanics, then, it is clear, if the pores of the filter extend straight through from outside to inside they would offer almost no resistance to the passage, at any rate, of the bacilli and germs of typhoid, but fortunately, as can be determined by a microscopical examination of a section of a Pasteur tube, the pores are sinuous and the windings which any organic particles passing through them must undergo seems to delay for a while their appearance on the inside of the tube. The few thus arrested may multiply in the pores of the tube until finally together with those brought by the throughflowing current the pores may become filled. When this happens the effluent will certainly show the presence of the objectionable germs, whatever they may be. The Pasteur filter, then, while probably efficient when used under skilful supervision in laboratories, cannot be relied upon to furnish sterile water in private houses, where in the majority of cases the skilful supervision will be limited to that of the servant girl.

In continuous filtration on a large scale abroad, it has been found by experience that the purification takes place almost entirely at the surface, where, before any special improvement of the effluent can take place, there must gather a felt-like bacteria jelly which is found to be the effective agent of filtration. With waters of ordinary impurity the surface of the sand becomes so far clogged in about a month's work as to require cleaning, and following which operation there succeeds a period, while a new jelly is forming, during which the improvement of the effluent is only slight. Light appears essential for the formation of this jelly in its greatest perfection, and thus far it has not been specially observed in the smaller filters used for house service here. An examination of a number of such has indicated the penetration of the organic impurities some distance into the sand in the manner already described.

The existence of the bacteria jelly on the surfaces of large continuous sand filters abroad has, however, led to a curious fad in filtration which is, so far as the present speaker knows, purely American—namely, the notion that the use of an alumina jelly in mechanical pressure filters furnishes the indispensable condition for perfect filtration. A discussion of this particular view of filtration would lead too far away from the present subject, and it is only referred to at all as an illustration of one of the essentially erroneous views of filtration which obtained considerable currency before the theory of the purification of water by filtration was as well understood as at the present time.

